# Synergistic effect of CART (cocaine- and amphetamine-regulated transcript) peptide and cholecystokinin on food intake regulation in lean mice

**DOI:** 10.1186/1471-2202-9-101

**Published:** 2008-10-21

**Authors:** Lenka Maletínská, Jana Maixnerová, Resha Matyšková, Renata Haugvicová, Zdeno Pirník, Alexander Kiss, Blanka Železná

**Affiliations:** 1 Institute of Organic Chemistry and Biochemistry, Academy of Sciences of the Czech Republic, Flemingovo nám. 2, 16610 Prague 6, Czech Republic; 2 Institute of Microbiology, Academy of Sciences of the Czech Republic, Vídeňská 1083, 14200 Prague 4, Czech Republic; 3 Institute of Experimental Endocrinology, Slovak Academy of Sciences, Vlárska 3, 83306 Bratislava, Slovak Republic

## Abstract

**Background:**

CART (cocaine- and amphetamine-regulated transcript) peptide and cholecystokinin (CCK) are neuromodulators involved in feeding behavior. This study is based on previously found synergistic effect of leptin and CCK on food intake and our hypothesis on a co-operation of the CART peptide and CCK in food intake regulation and Fos activation in their common targets, the nucleus tractus solitarii of the brainstem (NTS), the paraventricular nucleus (PVN), and the dorsomedial nucleus (DMH) of the hypothalamus.

**Results:**

In fasted C57BL/6 mice, the anorexigenic effect of CART(61-102) in the doses of 0.1 or 0.5 μg/mouse was significantly enhanced by low doses of CCK-8 of 0.4 or 4 μg/kg, while 1 mg/kg dose of CCK-A receptor antagonist devazepide blocked the effect of CART(61-102) on food intake. After simultaneous administration of 0.1 μg/mouse CART(61-102) and of 4 μg/kg of CCK-8, the number of Fos-positive neurons in NTS, PVN, and DMH was significantly higher than after administration of each particular peptide. Besides, CART(61-102) and CCK-8 showed an additive effect on inhibition of the locomotor activity of mice in an open field test.

**Conclusion:**

The synergistic and long-lasting effect of the CART peptide and CCK on food intake and their additive effect on Fos immunoreactivity in their common targets suggest a co-operative action of CART peptide and CCK which could be related to synergistic effect of leptin on CCK satiety.

## Background

Information on the metabolic status of the organism enters and is processed in the hypothalamus and in the nucleus tractus solitarii (NTS) of the brainstem (hindbrain). In the hypothalamic arcuate nucleus (ARC), adiposity signal leptin influences expression of peptides affecting food intake such as anorexigenic cocaine- and amphetamine-regulated transcript (CART). ARC neurons project to other hypothalamic areas such as the paraventricular nucleus (PVN) and the lateral hypothalamic area (LHA) (for reviews, see [1-5]). Both PVN and LHA convey neuronal signals to the brainstem where they are integrated with afferent input of cholecystokinin (CCK) [2], satiety peptide of gut origin. For the satiety effect of CCK, leptin signaling in ARC was found necessary [6]. Recently, CCK was shown to facilitate access of leptin to hypothalamic areas and modulate body weight [7].

Satiety effect of CCK is mediated by cholecystokinin A (CCK-A or CCK-1) receptors [8] expressed abundantly not only in the brainstem but also in the hypothalamus [9,10]. Unlike CCK receptors, receptors of CART peptide have not been found yet despite of a well-known anorexigenic effect of CART [3,11,12] and its stimulating effect on anxiety-like reactions [13] or analgesia [14]. Analogously, CART receptor antagonists have not been designed yet.

After its peripheral administration, CCK affected neuronal activity particularly in NTS, the area postrema, the locus coeruleus, PVN, and the dorsomedial nucleus (DMH) [5,15-18]. Similarly, injection of the CART peptide either into the third, fourth, or the lateral ventricle suppressed food intake [11,12,14,19,20] and stimulated expression of c-Fos in NTS, the parabranchial nucleus, PVN and DMH [21,22].

Co-localization of CART peptide and CCK-A receptor in vagal afferent neurons suggested that CART peptide might take part in mediating satiety effects of cholecystokinin [23]. Interestingly, a lowered leptin level after 48- hour food deprivation affected expression neither of CCK-A receptor [23] nor CART in nodose ganglion neurons [24]. However, recently, CART expression in rat vagal afferent neurons was found negligible after 24-hour fasting, up-regulated by CCK, and restored after re-feeding. The action of CCK on CART expression was shown to be mediated by activation of protein kinase C and cAMP response element binding protein (CREB) and was inhibited by orexigenic ghrelin [25].

Relationship between CCK and the CART peptide was documented also at pancreatic exocrine secretion of amylase where the stimulating effect of CART peptide was inhibited *in vivo *but not *in vitro *by CCK-A receptor antagonist devazepide [26].

Finally, a synergistic anorexigenic effect of CCK and CART peptide was suggested in goldfish [27], but no experimental details were given.

Data on interaction of CART peptide with other peptides regulating food intake have been scarce up to now. Besides the well-known suppression of the orexigenic effect of NPY by CART peptide [11,28], CART peptide-induced hypophagia and brain c-Fos expression was prevented by blocking central receptors for glucagon-like peptide 1 (GLP-1) [29].

The previously described findings point to a neurochemical link between CART peptide and CCK with regard to a previously found synergistic effect of leptin and CCK on food intake [15]. Therefore, in the present study, the idea is proposed that a co-operative action of the CART peptide and CCK might be involved in communication between ARC and NTS. Potential cooperation between central CART peptide and peripheral CCK in the short-term regulation of food intake in lean mice was investigated. To compare neuronal activation after administration of CART peptide, CCK, or simultaneous administration of CART peptide and CCK, c-Fos activation in three important brain areas involved in food intake regulation, PVN, DMH, and NTS, was also determined. In addition, the exploratory behavior of mice after administration of the above-mentioned compounds is described, which is an important element complementing food intake data.

## Methods

### Materials

Cholecystokinin octapeptide (CCK-8, Asp-Tyr(SO_3_H)-Met-Gly-Trp-Met-Asp-Phe-NH_2_, NeoMPS, Strasbourg, France), CCK-A receptor antagonist devazepide (L364,718) or CCK-B receptor antagonist L365,260 (gift from ML Laboratories, Liverpool, UK) and CART(61-102) (Bachem, Bubendorf, Switzerland) were used in the experiments.

The Fos (No 94012) antiserum was kindly provided by Dr. J.D. Mikkelsen (NeuroSearch A/S Ballerup, Denmark). The specificity and sensitivity of Fos antiserum have already been tested previously [30].

### Experimental animals

Male C57BL/6 mice obtained from the Institute of Molecular Genetics (Prague, Czech Republic) were housed in standard conditions (temperature of 23°C, daily cycle of 12 h light and dark (light from 6:00)). They were given *ad libitum *water and standard chow diet (St-1, Velaz, Koleè, Czech Republic). They were 14–16 weeks old (25–30 g) when cannulation and following experiments were performed. All experiments followed the ethical guidelines for animal experiments and the Act of the Czech Republic Nr. 246/1992 and were approved by the Committee for experiments with laboratory animals of the Academy of Sciences of the Czech Republic.

### Cannula placement

Mice were implanted with cannulas (Plastics One, Roanoke, USA) into the third ventricle (AP 2 mm, V 3 mm) as described earlier [31]. Thereafter, the animals were placed into separate cages with free access to food and water and allowed to recover from surgery at least seven days before starting the experiment.

### Food intake experiments

Before starting the food intake experiment, the mice were randomly divided into groups of 6–8 mice and were fasted overnight (17 h) with free access to water.

On the day of the experiment, the individual groups of mice underwent the following treatments before being given weighed food (at t = 0 around 8:30) and the registration of food intake started.

#### Experiment 1 Individual and combined administration of CCK-8 and CART(61-102)

Doses, route of administration and time schedule are described in Table 1.

**Table 1:** Administration of compounds in mice, in food intake Experiment 1 and 2

**Experiment 1**						
Compound	Dose i.p.	Time of application	Compound	Dose i.c.v.	Time of application	No. animals

saline		-20 min				8
CCK-8	0.4 μg/kg	-20 min				6
	4	-20 min				6
			saline		-15 min	8
			CART	0.1 μg/mouse	-15 min	6
				0.5	-15 min	6
CCK-8	0.4 μg/kg	-20 min	CART	0.1 μg/mouse	-15 min	6
	4	-20 min		0.1	-15 min	6
	0.4 μg/kg	-20 min		0.5 μg/mouse	-15 min	6
	4	-20 min		0.5	-15 min	7

**Experiment 2**						

devazepide	1 mg/kg	-45 min				6
	1	-45 min	CART	0.1 μg/mouse	-15 min	6
	1	-45 min		0.5	-15 min	6
L365,260	1 mg/kg	-45 min				6
	1	-45 min		0.5	-15 min	7

At time = 0 min, mice were given weighed food pellets.

#### Experiment 2 Individual and combined administration of CCK receptor antagonists and CART(61-102)

Doses, route of administration and time schedule are described in Table 1.

The volume of i.p. injected solutions was 0.2 ml/mouse, i.c.v. injected solutions were of 5 μl/mouse and were infused in 20 s using an infusion pump; the infusion cannula was left in place for a further 20 s to prevent reflux. Each animal was used only once, the experiment was repeated with a new set of mice.

At t = 0 min, i.e. 15 min after i.c.v. injection of CART(61-102) or 20 min after i.p. injected CCK-8 and 45 min after i.p. injection of devazepide or L365,260, mice were given weighed food pellets. The pellets were replaced with fresh ones every 30 min and weighed. Food intake was followed for 5 h. Animals had free access to water during the experiment. The results are expressed in grams of food consumed per mouse. The placement of the cannula was verified histologically.

### Fos Immunohistochemistry

#### Tissue processing

For Fos immunohistochemical processing, overnight fasted mice (n = 5 mice/group) were treated: a/i.c.v. with saline, b/i.p. with CCK-8 (0.4 μg/kg), c/i.c.v. with CART(61-102) (0.1 μg/mouse) or d/i.p. with CCK-8 followed by i.c.v. injected CART(61-102) (doses as described above). The time schedule of injections was identical as in the food intake study (Table 1). Sixty minutes after i.c.v. injection, the mice were deeply anesthetized with pentobarbital (50 mg/kg, i.p.) and perfused transcardially with 0.1 M phosphate buffer (PB, pH 7.4) containing 4% paraformaldehyde, 0.1% glutaraldehyde, and 10% picric acid (w/w). Then the brains were removed, postfixed in the same fixative overnight at 4°C, and infiltrated with 30% sucrose in 0.1 M PB for 48 h at 4°C. Before sectioning, the brains were rapidly (20 sec) frozen in cold isopentane (-30/-40°C) and placed into a Reichert cryocut device adjusted to -16°C for 1 h. Location of i.c.v. cannulas in the third ventricle was verified during sectioning and 30 μm coronal sections were cut from the brains and collected as free floating in cold (4°C) PB.

#### Immunohistochemistry

Free floating sections were repeatedly washed in cold PB followed by preincubation in 3% H_2_O_2 _for 40 min at room temperature. They were incubated with polyclonal Fos protein antiserum (1:2000), diluted in 0.1 M PB containing 4% normal goat serum (Gibco, Grand Island, NY, USA), 0.5% Triton X-100 (Koch-Light Lab. Ltd., Colnbrook, Berks, England), and 0.1% sodium azide for 48 h at 4°C. After several rinses in PB, the sections were incubated with biotinylated goat-anti-rabbit IgG (1:500, VectorStain Elite ABC, Vector Lab., Burlingame, CA, USA) for 90 min at room temperature. Next PB rinses were followed by incubation with the avidin-biotin peroxidase complex (1:250) for 90 min at room temperature. PB washing was followed by washing in 0.05 M sodium acetate buffer (SAB, pH 6.0). The Fos antigenic sites were visualized with 0.0266% 3,3'-diaminobenzidine tetrahydrochloride (DAB) dissolved in SAB containing 0.0042% H_2_O_2 _and 2.5% nickel ammonium sulfate, for 7 min. The metal-intensification of DAB produced black staining in the labeled nuclei. Finally, the sections were rinsed in 0.05 M SAB, mounted into 0.1% of gelatine dissolved in 0.0125 M SAB, air-dried and coverslipped with Permount (Sigma, St. Louis, MO, USA). Immunostaining of negative control, which did not show any antiserum immunolabeling, included substitution of the primary antiserum with normal rabbit serum, and sequential elimination of the primary or secondary antibody from the staining series.

#### Evaluation of the immunostaining

An identical set of mice was used for determination of Fos immunoreactivity in NTS, PVN, and DMH. Counting of Fos immunoreactive cells within the NTS, from Bregma -7.48 mm to Bregma -7.32 mm, PVN, from Bregma -0.7 mm to -0.94 mm, and DMH, from Bregma -1.46 mm to – 1.82 mm according to the mouse brain atlas [32], was performed separately in each side of the sections. Quantitative assessment was performed from the images captured with a Canon digital camera (PowerShot S40) and Leica DMLS light microscope in a computer screen obtained from 5–6 brain sections per animal. Representative sections were captured by the same computerized system. The counting of Fos-positive neurons was done by one of the authors under blinded conditions (the counted slides from each animal were analyzed independently and randomly and encoded by other person).

### Open field locomotor activity

Locomotor activity was measured using the VideoMot system (TSE Systems, Bad Homburg, Germany). *Ad libitum *fed mice were placed individually in the open field (1 × 1 m) and their locomotor activity, i.e. total distance traveled, was measured for 10 min. Mice were administered with i.p. injection of CCK-8 (4 μg/kg), i.p. injection of devazepide (1 mg/kg), i.c.v. administration of CART(61-102) (0.1 and 0.5 μg/mouse) or their combination with a time schedule as described in feeding experiments in Table 1 (n = 5 mice/group).

### Statistics

Data are presented as means ± SEM for the number of animals indicated in the Figures. They were analyzed by the non-repeated measures one-way analysis of variance (ANOVA) followed by Bonferroni *post hoc *test using Graph-Pad Software (San Diego, CA, USA). P < 0.05 was considered statistically significant.

## Results

### Synergistic action of CART peptide and CCK-8 in food intake of fasted lean mice

#### Experiment 1

**Figure 1:**
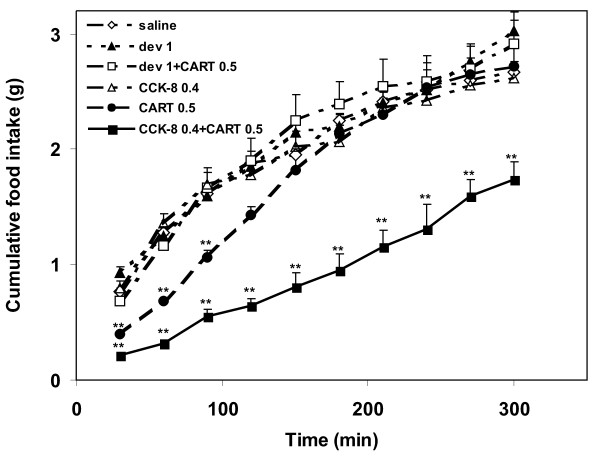
**Cumulative food intake **of 17-h fasted mice after administration of CCK-8 (0.4 μg/kg, i.p.), devazepide (1 mg/kg, i.p.) or after administration of saline (i.c.v.), CART(61-102) (0.5 μg/mouse, i.c.v.), CCK-8 (0.4 μg/kg, i.p.) followed by CART(61-102) (0.5 μg/mouse, i.c.v.), and devazepide (1 mg/kg, i.p.) followed by CART(61-102) (0.5 μg/mouse, i.c.v.). At time 0, weighed food was given to mice. Food intake is expressed in grams of food consumed (n = 6–8 mice per group). ** P < 0.01 vs. saline-treated group [ANOVA, F_4,39 _= 5.92].

Cumulative food intake of male C57BL/6 mice was measured for all doses of compounds described in Methods (Table 1) and is illustrated by curves in Fig. 1. The feeding response to saIine injected i.p. or i.c.v. did not show any significant difference.

**Figure 2:**
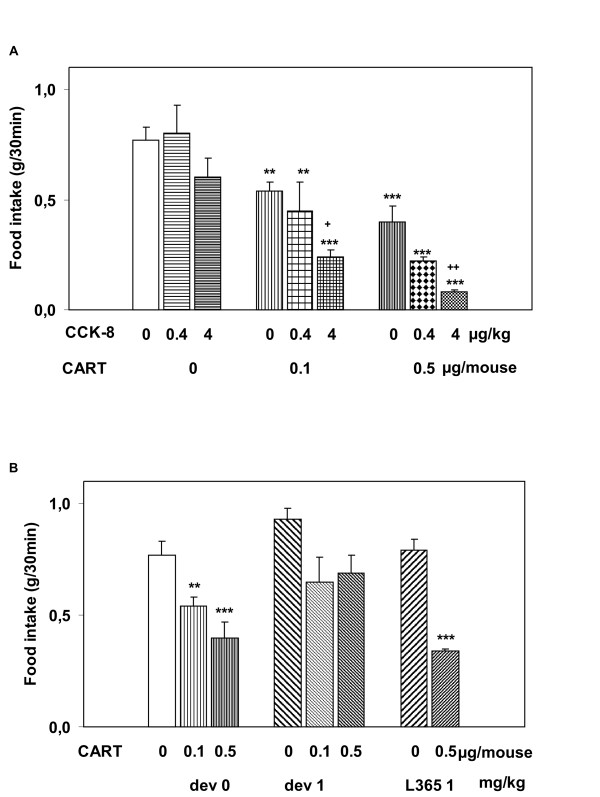
**Dose-related inhibition of food intake **in 17-h fasted mice after administration of: A/ CCK-8 (0.4 and 4 μg/kg, i.p.) or i.c.v. saline, CART(61-102) (0.1 and 0.5 μg/mouse, i.c.v.), CCK-8 (0.4 and 4 μg/kg, i.p.) plus CART(61-102) (0.1 and 0.5 μg/mouse, i.c.v.), or B/ i.p. devazepide (1 mg/kg) alone or followed by i.c.v. CART(61-102) (0.1 and 0.5 μg/mouse) and i.p. L365,260 (1 mg/kg) alone or followed by i.c.v. CART(61-102) (0.5 μg/mouse). Food intake is expressed in grams of food consumed in the first 30 min after presentation of food (n = 6–8 mice per group). ** P < 0.01, *** P < 0.001 vs. saline-treated group, ^+ ^P < 0.05 vs. CART(61-102) 0.1 μg/mouse, ^++ ^P < 0.05 vs. CART(61-102) 0.5 μg/mouse [ANOVA, F_12,78 _= 13.88]. CART – CART(61-102), dev – devazepide, L365 – L365,260.

CART(61-102) at a dose of 0.5 μg/mouse significantly attenuated food intake up to 105 min after its i.c.v. injection with maximum between 45 and 75 min after i.c.v. injection, i.e. after the first and second measurements of food intake (Fig. 1). A dose-dependent anorexigenic effect of CART(61-102) is obvious in Fig. 2a. In C57BL/6 fasted mice, CCK-8 doses of 0.4 and 4 μg/kg i.p. did not significantly modify food intake (Fig. 2a).

Simultaneous application of CCK-8 and CART(61-102) reduced food intake more significantly than each single peptide (Fig. 1, 2a), and the effect lasted for more than five hours (Fig. 1). Doses of CCK-8 (0.4 and 4 μg/kg) increased the anorexigenic effect of CART(61-102) in a dose-related way (Fig. 2a). Table 2 summarizes values of food intake at the time of maximal effect of administered peptides (30 minutes after addition of pellets).

**Table 2:** Food intake of 17-h fasted mice during first 30 min measured (maximal effect) in Experiment 1 and 2

Compound/dose				Food intake/30 min (g ± SEM)
i.p.		i.c.v.		
saline				0.77 ± 0.06
		saline		0.73 ± 0.07

CCK-8	0.4 μg/kg			0.80 ± 0.13
	4			0.60 ± 0.09
		CART	0.1 μg/mouse	0.54 ± 0.04 **
			0.5	0.40 ± 0.07 ***
CCK-8	0.4 μg/kg	CART	0.1 μg/mouse	0.45 ± 0.13 **
	4		0.1	0.24 ± 0.03 *** ^+^
CCK-8	0.4 μg/kg	CART	0.5 μg/mouse	0.22 ± 0.02 ***
	4		0.5	0.08 ± 0.01 *** ^++^

devazepide	1 mg/kg			0.93 ± 0.05
	1	CART	0.1 μg/mouse	0.65 ± 0.11
	1		0.5	0.69 ± 0.08
L365,260	1			0.79 ± 0.05
	1		0.5	0.34 ± 0.01 ***

**P < 0.01, ***P < 0.001 versus saline-treated group. ^+^P < 0.05 versus group treated by CART, 0.1 μg/mouse, ^++ ^P < 0.01 versus group treated by CART, 0.5 μg/mouse.

#### Experiment 2

CCK-A receptor inactivation with its antagonist devazepide prevented the anorexigenic effect of CART(61-102) (0.1 and 0.5 μg/mouse), whereas the specific CCK-B receptor antagonist L-365,260 did not affect the CART-induced decrease in food intake (Fig. 2b, Table 2).

### Fos immunoreactivity in NTS, PVN, and DMH after CART peptide and CCK administration

**Figure 3:**
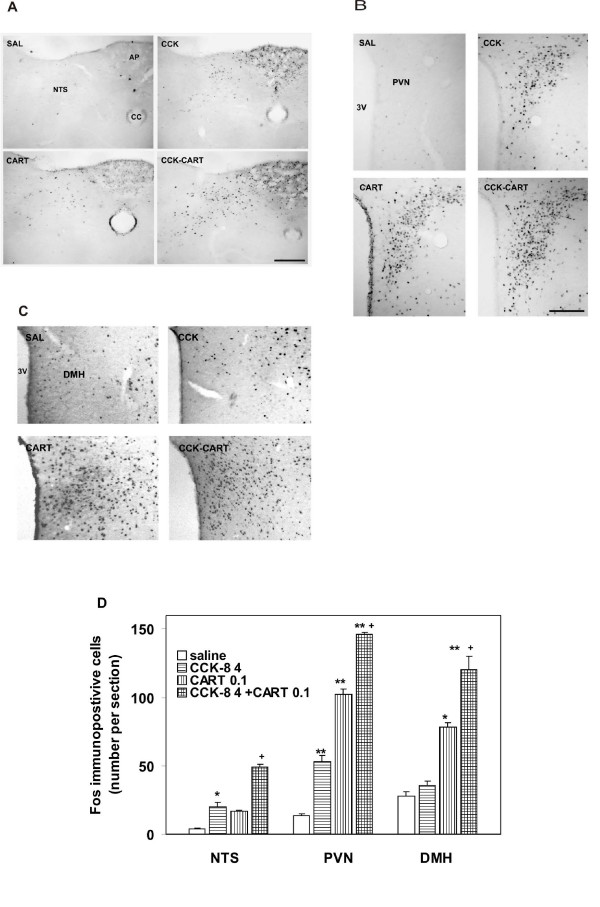
**Fos immunoreactivity**: Fos-immunostained cells in coronal section of A/ NTS, B/ PVN, C/ DMH, D/ Number of Fos-immunopositive cells in NTS, PVN and DMH 60 minutes after i.c.v. application of saline (SAL), CCK-8 (4 μg/kg, i.p.), CART(61-102) (0.1 μg/mouse, i.c.v.), and their combination expressed unilaterally per animal (n = 4–5) and section (n = 5–6). ^+ ^P < 0.01 vs. SAL, CCK and CART, * P < 0.05, ** P < 0.01 vs. SAL [ANOVA, for NTS F_3,16 _= 26.23, for PVN F_3,13 _= 100.35, for DMH F_3,12 _= 58.19]. SAL – saline, NTS – solitary tract nucleus, CC – central canal, AP – area postrema, PVN – paraventricular nucleus. Bar = 100 μm.

The doses of CCK-8 (4 μg/kg) and CART(61-102) (0.1 μg/mouse) used by us for determination of Fos immunoreactivity correspond to those used previously by others [18,19,30]. Statistical analysis by one way ANOVA documented the impact of CCK-8, CART(61-102) as well as their combined treatment on the number of Fos immunopositive cells both in NTS (F_3,16 _= 26.23, p < 0.000001), PVN (F_3,13 _= 100.35, p < 0.000001), and DMH (F_3.12 _= 58.19, p < 0.000001) in lean C57BL/6 mice (Fig. 3).

In NTS, the Fos immunoreactivity in saline-treated controls was minimal and sporadic (Fig. 3a). The number of Fos immunoreactive cells distinctly increased after i.p. application of CCK-8 (0.4 μg/kg) compared with saline-treated mice (P < 0.05) (Fig. 3a, d). A similar, but statistically insignificant increase was observed after i.c.v. infusion of CART(61-102) (0.1 μg/mouse). The *post hoc *test showed a significant effect (P < 0.01) of parallel administration of CCK-8 and CART peptide on the Fos activation of cells in NTS in comparison with all other groups of animals (Fig. 3a, d).

In PVN and DMH, Fos immunoreactivity followed a similar trend as in the NTS. In control animals, a weak Fos signal was found (Fig. 3b,c). A significant increase in Fos immunoreactivity was registered after application of CCK-8 (p < 0.01), CART(61-102) (p < 0.01), and parallel administration of CCK-8 and CART(61-102) (p < 0.01) in comparison with saline-treated mice (Fig. 3b, d). The *post hoc *test revealed a significantly higher increase in the number of Fos-immunopositive cells in PVN and DMH after CART(61-102) application compared with application of CCK-8 (Fig. 3b, c); simultaneous application of the above-mentioned peptides (p < 0.01) activated Fos in PVN and DMH more significantly than each particular peptide alone (p < 0.01).

### Behavioral effect after parallel CART peptide and CCK-8 injection

**Figure 4:**
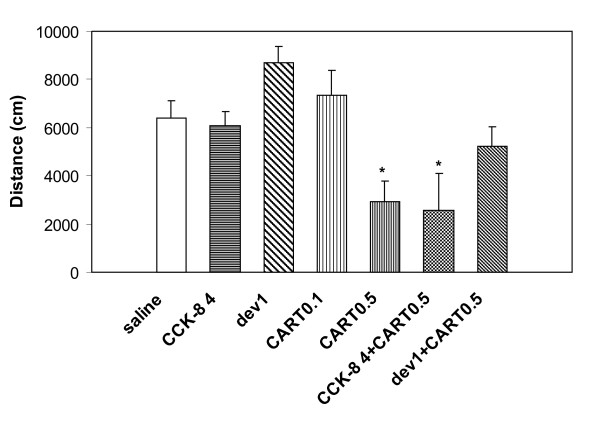
**Behavioral activity **of fed mice (open field test, total distance traveled for 10 min) after i.p. administration of CCK-8 (4 μg/kg) or devazepide (1 mg/kg), after i.c.v. administration of saline, CART(61-102) (0.1 and 0.5 μg/mouse), or after combined injection of i.p. CCK-8 or devazepide followed by i.c.v. CART(61-102). ** P < 0.01 vs. saline-treated group [ANOVA, F_6,38 _= 6.29]. CART – CART(61-102), dev – devazepide, L365 – L365,260.

Open field locomotor activity of fed lean mice was measured for 10 min after i.c.v. administration of CART(61-102), i.p. administration of CCK-8 and devazepide or their combination (see Methods). The goal was to find out whether in behavioral tests, the additive effect of CART peptide and CCK-8 occurred similarly as in the food intake experiments. The results clearly showed (Fig. 4) that the CART peptide (0.5 μg/mouse) alone or in combination with CCK-8 (4 μg/kg) significantly shortened the distance traveled in the open field compared to that of the saline-treated group. The decrease of locomotor activity after CART peptide treatment was reversed by CCK-A antagonist devazepide (Fig. 4).

## Discussion

In this study, the effect of simultaneous administration of CART peptide and CCK on food intake, Fos immunoreactivity in NTS, PVN, and DMH and locomotor activity points to a positive cooperation between the CART peptide and CCK.

Intracerebroventricular injections of CART(61-102) into the third ventricle of fasted C57BL/6 mice attenuated food intake (Fig. 1, 2) in a dose-dependent manner similarly as in the studies of Thim *et al*. [12], Bannon *et al*. [14] and Vrang *et al*. [22]. At the doses of 0.4 and 4 μg/kg, CCK-8 did not show any significant effect on food intake in fasted C57BL/6 mice (Fig. 2a) (with EC_50 _about 15 μg/kg determined in a preliminary study), unlike fasted outbred NMRI mice that were sensitive to CCK-8 dose of 4 μg/kg (with EC_50 _about 9 μg/kg [31]). Serum leptin after 17 h fasting was 50% higher in the robust NMRI mice compared to the subtle C57BL/6 mice (0.45 ± 0.14 ng/ml in NMRI [33] and 0.30 ± 0.06 ng/ml in C57BL/6 male mice, our non-published data), which was probably the reason why the threshold dose for CCK-8 was higher in C57BL/6 than in NMRI mice (4 versus 0.4 μg/kg, respectively).

The 48-hour long fasting was shown to attenuate the satiety response to CCK because of a lowered leptin level [34]. Barrachina *et al*. [15] demonstrated that leptin cooperated synergistically with satiety effect of CCK in 24-hour fasted C57BL/6 mice [15]. Therefore in this study, it is supposed that synergistic co-operation of leptin and CCK was preserved in 17- hour fasted C57BL/6 mice despite lowered leptin level after fasting.

In this study, low doses of CCK enhanced the anorexigenic effect of CART peptide in a dose-dependent way and prolonged the time of the CART peptide effect (Fig. 1) that was in agreement with our hypothesis on a synergistic effect of CART(61-102) and CCK-8 on food intake (Fig. 2a). A direct involvement of CCK-A receptor in the anorexigenic effect of CART peptide was demonstrated in this study, because selective CCK-A receptor antagonist devazepide [8] was found to block the effect of CART peptide on food intake (Fig. 2b). Similarly, the anorexigenic action of CART peptide in rats was prevented by GLP-1 receptor antagonist [29]. The fact that L365,260, selective CCK-B receptor antagonist, which is not involved in food intake regulation [8], did not alter the decrease in food intake induced by the CART peptide (Fig. 2b) supported the idea of the involvement of exclusively CCK-A receptor type in its co-operation with the CART peptide.

Co-administration leptin with CCK-8 in a dose of 3.5 μg/kg i.p. that was considered subthreshold potentiated satiety and activation of brainstem neurons in lean C57BL/6 mice and also enhanced the number of Fos-positive neurons in PVN [15]. In this study, the Fos immunoreactivity after injection of the CART peptide or/and CCK-8 was determined in NTS, PVN, and DMH, common targets of CART peptide [21,22] and CCK [5,15-18], in the identical set of mice. A strong increase in the number of Fos-positive cells was found (Fig. 3) when the dose of CART peptide that significantly attenuated food intake was co-administered with a dose of CCK-8 that did not influence food intake; this response was more significant than the response to each particular peptide. This result confirmed the co-operation between the CART peptide and CCK-8 in the brainstem and hypothalamic PVN and DMH, where satiety signals originating in the periphery as well as in the forebrain are processed. PVN is highly innervated by afferent projections from DMH. It was shown that CCK might activate corticotropin-releasing factor (CRF) neurons in DMH [18] via noradrenergic projections from NTS [35]. This pathway could contribute to the additive effect of CCK and CART peptide in the DMH.

Finally, behavioral data followed the trend of the feeding tests. CART peptides are known to influence locomotor activity and analgesia [13,14]. However, the significance of the effects differed in different studies. In this study, a dose of CCK-8 that did not attenuate food intake (4 μg/kg i.p.) did not also significantly lower the locomotor activity of C57BL/6 mice in the open field test (Fig. 4), while CART(61-102) at a dose of 0.5 μg/mouse and its combination with CCK-8 significantly suppressed the distance traveled in open field test. Analogously, devazepide alone did not significantly affect the locomotor activity of mice compared to the saline-treated group but blocked the effect of CART peptide (Fig. 4). These findings again suggest that the effect of CART peptide was pronounced through activation of CCK-A receptor and point to satiety-related sedation. Analgesic hot-plate test was also performed, but none of the above-mentioned compounds significantly changed the paw-licking latency compared to the saline-treated group (data not shown).

The long lasting effect of synergistic action of CCK and the CART peptide in this study could be interpreted according to de Lartigue *et al*. [25] as a result of CCK- induced expression of CART and a consequent effect of the endogenously produced CART peptide after the exogenous CART peptide was exhausted. On the other hand, Broberger *et al*. described that expression of CCK-A receptor does not depend on the metabolic status [23].

The integratory role of the ARC-PVN-DVC (dorsal vagal complex in which NTS is included) was suggested for the synergistic action of leptin and CCK on the attenuation of food intake and body weight [15]. Food intake was affected by parallel administration of leptin and CCK more potently than by individual application of the hormones and lasted at least 7 h. Leptin and CCK activation pathways appeared to overlap at three discrete nuclei: the PVN, parabrachial nucleus and NTS [5].

## Conclusion

In this study, the synergistic effect of CCK and CART peptide on food intake targeted hypothalamic PVN and DMH and NTS, which suggested that CART peptide might act as a mediator of the leptin anorexigenic effect both in the hypothalamus and brainstem. Identification of the CART peptide receptor and designing its antagonist is necessary for identification of further possible cooperative actions of the CART peptide.

## Authors' contributions

LM and BŽ designed the study, drafted the manuscript and performed the statistical analysis. LM, JM, RM and BŽ carried out the majority of the experiments and data collection. RH participated in implantation of cannulas. ZP and AK performed immunohistochemistry. All authors read and approved the final manuscript.
